# Investigating the Impact of *Fusobacterium nucleatum* on Oxidative Stress, Chemoresistance, and Inflammation in Inflammatory Bowel Disease and Colorectal Cancer: Rationale and Design of a Clinical Trial

**DOI:** 10.3390/ijms26167823

**Published:** 2025-08-13

**Authors:** Pierluigi Consolo, Carlotta Giorgi, Concetta Crisafulli, Francesco Fiorica, Paolo Pinton, Nicola Maurea, Sonia Missiroli, Vincenzo Quagliariello, Beatrice Mantoan, Alessandro Ottaiano, Giovanni Francesco Pellicanò, Germano Orrù, Alessandra Scano, Irene Cacciola, Teresa Pollicino, Giordana Di Mauro, Salvatore Berretta, Alessia Bignucolo, Enrica Toscano, Giuliana Ciappina, Massimiliano Berretta

**Affiliations:** 1 Endoscopy Digestive Unit, Department of Clinical and Experimental Medicine, G. Martino University Hospital, University of Messina, 98122 Messina, Italy; pconsolo@unime.it (P.C.); concetta.crisafulli@unime.it (C.C.); 2 Department of Medical Sciences, University of Ferrara, 44121 Ferrara, Italy; carlotta.giorgi@unife.it (C.G.); paolo.pinton@unife.it (P.P.); sonia.missiroli@unife.it (S.M.); giuliana.ciappina@unife.it (G.C.); 3 Department of Clinical Oncology, AULSS 9 Scaligera, 37122 Verona, Italy; francesco.fiorica@aulss9.veneto.it (F.F.); beatrice.mantoan@aulss9.veneto.it (B.M.); 4 Division of Cardiology, Istituto Nazionale Tumori-IRCCS Fondazione Pascale, 80131 Napoli, Italy; n.maurea@istitutotumori.na.it (N.M.); quagliariello.enzo@gmail.com (V.Q.); 5 Istituto Nazionale Tumori di Napoli, IRCCS “G. Pascale”, Via M. Semmola, 80131 Naples, Italy; a.ottaiano@istitutotumori.na.it; 6 Unit of Infectious Diseases, Department of Clinical and Experimental Medicine, G. Martino University Hospital, University of Messina, 98122 Messina, Italy; giovanni.pellicano@unime.it; 7 Molecular Biology Laboratory, Department of Surgical Sciences, University of Cagliari, 09124 Cagliari, Italy; orru@unica.it (G.O.); alessandra.scano77@unica.it (A.S.); 8 Department of Clinical and Experimental Medicine, G. Martino University Hospital, University of Messina, 98122 Messina, Italy; irene.cacciola@unime.it (I.C.); teresa.pollicino@unime.it (T.P.); alessia.bignucolo@unime.it (A.B.); 9 School of Specialization in Medical Oncology, Department of Human Pathology “G. Barresi”, G. Martino University Hospital, University of Messina, 98122 Messina, Italy; giordana.di.mauro@studenti.unime.it (G.D.M.); enrica.toscano@studenti.unime.it (E.T.); 10 Department of Human Pathology “G. Barresi”, G. Martino University Hospital, University of Messina, 98122 Messina, Italy; salvatore.berretta@studenti.unime.it; 11 Division of Medical Oncology, G. Martino University Hospital, University of Messina, 98124 Messina, Italy

**Keywords:** microbiota, IBD, CRC, oxidative stress, ROS, NOS, lipid peroxidation, autophagy, chemoresistance, prognostic factor, cancer cell death, host immune responses

## Abstract

*Fusobacterium nucleatum* (*F. nucleatum*), a Gram-negative anaerobe, is increasingly implicated in the pathogenesis of colorectal cancer (CRC) and inflammatory bowel disease (IBD). Its adhesin FadA enables epithelial adherence and invasion, promoting inflammation and tumorigenesis. *F. nucleatum* has been shown to activate the NLRP3 inflammasome, leading to IL-1β release, and is associated with chemoresistance and poor prognosis in CRC. Additionally, lipid peroxidation markers such as malondialdehyde (MDA) and 4-hydroxy-nonenal (4-HNA) may contribute to inflammation-driven carcinogenesis. This study protocol aims to investigate the role of *F. nucleatum* in the development and progression of IBD and CRC through integrated clinical, molecular, and imaging approaches. The protocol involves quantifying *F. nucleatum* in tissue biopsies across disease stages and assessing correlations with inflammatory and oxidative markers. It will explore the bacterium’s involvement in NLRP3 inflammasome activation, IL-1β production, and autophagy, and its potential contribution to chemoresistance. Furthermore, radiomic analysis of computed tomography (CT) images will be performed to identify imaging phenotypes associated with microbial load and inflammatory activity. Although primarily a protocol, the study includes preliminary in vitro data showing that exposure to FadA significantly increases inflammatory markers in Caco-2 cells, supporting the hypothesis that *F. nucleatum* contributes to a pro-inflammatory, pro-tumorigenic microenvironment relevant to CRC progression.

## 1. Introduction

Inflammatory bowel disease (IBD) and colorectal cancer (CRC) are two highly prevalent conditions and carry a substantial burden on public health [1]. The gut microbiota is a dynamic community of microorganisms residing in the gastrointestinal tract. It consists of diverse symbionts, including bacteria, archaea, eukarya, viruses, and parasites. The gut microbiota performs essential functions in the immunological, metabolic, structural, and neurological landscapes of the human body, affecting the host’s physiological homeostasis. [2,3]. Several studies have proposed a model of CRC as a complex ecosystem composed not only of neoplastic cells but also of microorganisms that can modulate tumor cell behavior. This microbial community, commonly referred to as the intratumoral microbiota, is also capable of interacting with the gut microbiota through mechanisms that remain incompletely understood [4].

The relationship between gut microbes and colon cancer involves complex interactions between the host’s immune system, metabolic pathways, and bacterial communities. Multiple studies have demonstrated that an impairment of the microbiota’s health status, known as dysbiosis, can promote a pro-inflammatory environment in the colon; this inflammation can lead to DNA damage in colonic cells and contribute to carcinogenesis [5,6].

The gut microbiota produces various metabolites that influence colon health. These include short-chain fatty acids (SCFAs) like butyrate and propionate that are typically protective, promoting healthy epithelial cells, reducing inflammation, and supporting the gut barrier function. However, dysbiosis can reduce SCFA production, leading to a compromised intestinal environment. Secondary bile acids (e.g., deoxycholic acid), produced by microbial metabolism of primary bile acids, can promote carcinogenesis by causing DNA damage and inflammation [7]. Microbiota-mediated inflammation can disrupt the intestinal epithelial barrier, allowing for bacterial translocation, and can increase the production of pro-inflammatory cytokines, which enhance the risk of DNA mutations in colon cells. Recent research highlighted the role of the microbiota in CRC recurrence; in fact, after initial treatment, the gut microbiota can continue to influence the tumor microenvironment, affecting immune surveillance, tissue repair, and inflammation. In addition to inflammation, chronic dysbiosis has been associated with increased oxidative stress in the colonic microenvironment. Elevated levels of reactive oxygen species (ROS) can induce oxidative DNA damage and lipid peroxidation, contributing to mucosal injury and epithelial transformation. Clinically, lipid peroxidation products such as malondialdehyde (MDA) and 4-hydroxy-nonenal (4-HNA) have been detected in colonic tissues of patients with IBD and CRC, and are considered potential biomarkers of oxidative imbalance and disease progression [8].

**Figure 1:**
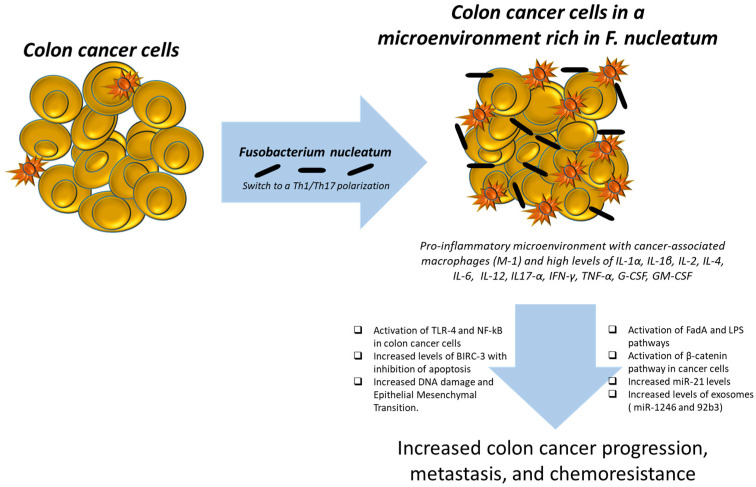
Key role of *F. nucleatum* in colon cancer progression, metastasis, and chemoresistance. In brief, *F. nucleatum* can change the colon cancer microenvironment in a pro-inflammatory fashion with a switch Th1–Th17 phenotype. *F. nucleatum*, through its FadA and lipopolysaccharide (LPS), increases β-catenin, miR-21, and oxidative stress in colon cancer cells. Moreover, this interaction leads to an increase in epithelial–mesenchymal transition, to a greater predisposition to DNA damage and secretion of cytokines and growth factors with anti-apoptotic and pro-metastatic potential.

Recent studies highlighted the role of specific microorganisms within the gut microbiota in promoting the development of CRC. For example, *Bacteroides fragilis* produces a toxin (BFT, *Bacteroides fragilis* toxin) that induces inflammation and DNA damage, leading to tumor formation [9]. Moreover, some strains of *E. coli* harbor virulent factors like the colibactin genotoxin, which can cause direct DNA damage in epithelial cells, triggering mutations that can lead to cancer. *Fusobacterium nucleatum* is well known for promoting colorectal tumorigenesis by increasing inflammation and immune evasion [10] (Figure 1).

Notably, some bacterial species can metabolize or modify chemotherapy drugs, altering their effectiveness. For instance, microbiota-mediated resistance to chemotherapy could make residual cancer cells harder to eliminate, thus increasing the risk of recurrence. Some microorganisms produce metabolites that enhance the efficacy of chemotherapy, while others may promote tumor cell survival and therapy resistance. Recent studies have demonstrated that immunotherapy outcomes can be modulated by the gut microbiota [11]. Research suggests that patients with a diverse and balanced microbiome respond better to certain immunotherapies. Conversely, dysbiosis can reduce the efficacy of treatments aimed at stimulating the immune system to attack cancer cells, potentially increasing the risk of recurrence [12].

## 2. The Pro-Oxidative Impact of *Fusobacterium nucleatum*: Preliminary Findings

*F. nucleatum*, a Gram-negative anaerobic bacterium, is an oral mycobiont associated with the development of periodontitis [13]. High levels of *F. nucleatum* have also been reported in CRC, where it is associated with poorer survival and the induction of resistance to chemotherapy [14,15]. *F. nucleatum* stimulates inflammation in gut mucosa and in murine macrophages by promoting NLPR3 inflammasome activation and IL-1ß secretion [16,17]. Furthermore, *F. nucleatum* drives autophagy activation in CRC cells [18]. Mechanistically, *F. nucleatum* targets the TLR4 and MYD88 innate immune signaling pathways, along with specific microRNAs, to activate the autophagy pathway, thereby altering the chemotherapeutic response in CRC. Autophagy, a conserved catabolic process responsible for the degradation and recycling of damaged cellular components, exerts a complex and context-dependent role in CRC. On one hand, autophagy maintains cellular homeostasis and prevents accumulation of oncogenic stress, thus acting as a tumor suppressor during early carcinogenesis. On the other hand, in established CRC, autophagy can support tumor cell survival under metabolic stress and therapeutic pressure, facilitating tumor progression and resistance to chemotherapy [19].

**Figure 2:**
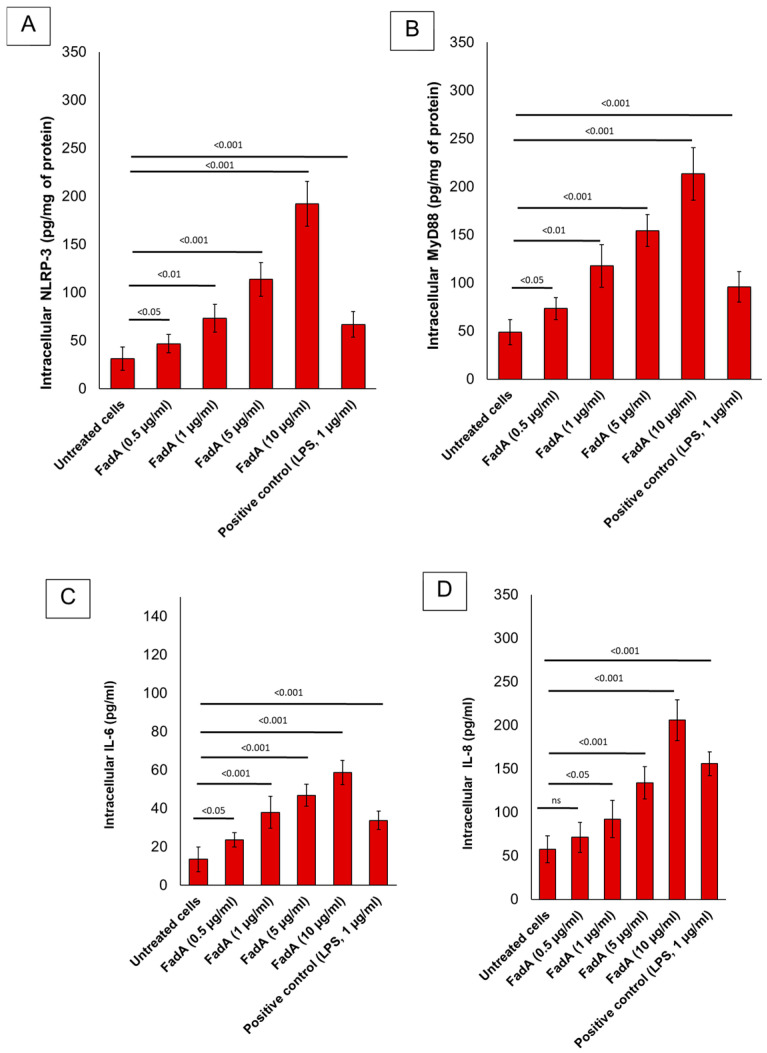
Intracellular levels of NLRP3 (**A**) and MyD88 (**B**), and secreted IL-6 (**C**) and IL-8 (**D**) in Caco-2 cells following FadA and LPS stimulation. Data are expressed as mean ± standard error (SE) from at least three independent experiments. Statistical significance: ns = not significant, *p* < 0.05, *p* < 0.01, *p* < 0.001 (ANOVA, post hoc Tukey’s test).

Consequently, *F. nucleatum* orchestrates an interconnected molecular network involving Toll-like receptors, microRNAs, and autophagy to influence CRC chemoresistance at the clinical, biological, and mechanistic levels [18]. In addition, an association of autophagy status with the amount of *F. nucleatum* in CRC has been proposed; in fact, the tumor expression of BECN1 (Beclin 1), a key positive regulator of autophagy, is inversely associated with the amount of *F. nucleatum* in CRC tissue, suggesting a possible role for autophagy in the elimination of invasive microorganisms [20]. *F. nucleatum* attaches to, penetrates, and triggers both oncogenic and inflammatory pathways to promote the proliferation of CRC cells via its distinctive Fusobacterium adhesin A (Fad A). FadA interacts with E-cadherin, initiating β-catenin signaling and modulating inflammatory and tumorigenic responses. Recent studies have revealed that *F. nucleatum* can activate the NLRP3 inflammasome via its virulence factor FadA, leading to increased secretion of IL-1β, which contributes to chronic intestinal inflammation [21]. The NLRP3 inflammasome represents a critical cellular pathway intricately involved in CRC pathogenesis, with significant implications for tumor progression and therapeutic targeting. NLRP3, a multiprotein complex central to innate immunity, regulates the activation of caspase-1 and subsequent secretion of pro-inflammatory cytokines IL-1β and IL-18. In CRC, aberrant activation of NLRP3 can promote a pro-tumorigenic inflammatory microenvironment that facilitates cancer cell proliferation, invasion, and metastasis. Chronic inflammation is a well-established driver of colorectal tumorigenesis, and NLRP3-mediated inflammation contributes to this process by modulating the crosstalk between tumor cells and the immune milieu [22]. Preliminary data from our in vitro experiments (Figure 2) suggest that FadA significantly increases inflammatory markers in the adenocarcinoma cell line Caco-2, which supports the hypothesis that *F. nucleatum* increases pro-oxidative molecules involved in colon cancer division. In more detail, the human colorectal adenocarcinoma cell line Caco-2 (ATCC, USA) was cultured in Dulbecco’s Modified Eagle Medium (DMEM) (Sigma-Aldrich, Merck KGaA, Darmstadt, Germany) supplemented with 10% fetal bovine serum (FBS), 1% penicillin–streptomycin (100 U/mL penicillin, 100 µg/mL streptomycin), and 1% L-glutamine at 37 °C in a humidified 5% CO_2_ atmosphere. Cells were maintained in 75 cm^2^ flasks and passaged upon reaching 80–90% confluence using 0.25% trypsin-EDTA (Sigma-Aldrich, Merck KGaA, Darmstadt, Germany). For experiments, cells were seeded in six-well plates (2 × 10^5^ cells/well) and allowed to adhere 24 h prior to treatment. Recombinant FadA protein (purity > 95%, endotoxin-free, 10 µg/mL) was obtained from Sigma-Aldrich (Merck KGaA, Darmstadt, Germany). The protein was diluted in sterile phosphate-buffered saline (PBS) (Euroclone S.p.A., Pero, Milan, Italy) to achieve final concentrations of 0.5, 1, 5, and 10 µg/mL for cell stimulation. After 24 h of cell attachment, the culture medium was replaced with fresh DMEM with 1% FBS to reduce basal proliferation. Cells were then stimulated as follows:

○ Control (untreated): Cells were incubated with only 1% FBS medium.
○ FadA (0.5 µg/mL)
○ FadA (1 µg/mL)
○ FadA (5 µg/mL)
○ FadA (10 µg/mL)
○ Positive control (LPS, 1 µg/mL): Cells were stimulated with *E. coli* LPS as a general inflammation control.

Cells were incubated for 48 h at 37 °C in a 5% CO_2_ atmosphere. Supernatants were collected for cytokine analysis (IL-6, IL-8) via ELISA, and cells were harvested for NLRP-3 and Myd-88 ELISA methods. All data were analyzed using GraphPad Prism. Results are expressed as mean ± standard deviation (SD). Statistical significance was determined using one-way analysis of variance (ANOVA) followed by a post hoc Tukey’s test. A *p*-value < 0.05 was considered statistically significant. Notably, preliminary results (Figure 2) from Caco-2 cells treated with FadA indicated a dose-dependent increase in pro-inflammatory cytokine secretion, including IL-6 and IL-8. This response was most pronounced at 10 µg/mL, suggesting a potential role for FadA in promoting pro-tumorigenic pathways. The same behavior was seen for NLRP-3 and Myd-88 expression, whose intracellular levels were significantly increased in FadA treated cells vs. the untreated group (*p* < 0.001). Further analysis is ongoing to confirm these findings.

## 3. Hypothesis and Aims

Building upon these observations, we hypothesize that *F. nucleatum* contributes to colorectal carcinogenesis through the induction of NLRP3-mediated inflammation and dysregulated autophagy. These pathways may alter the gut microenvironment and facilitate cancer initiation and progression. In fact, *F. nucleatum* interacts with the CRC immune microenvironment, inducing macrophages to M2 polarization by activating TLR4/IL6/p-STAT3/c-Myc and NFKB/S100A9 signals [23]. Moreover, lipopolysaccharides of *F. nucleatum* significantly increased the secretion of pro-inflammatory cytokines IL-6, IL-1ß, TNF-a, IL-8, and PGE2 in macrophages [24]. Therefore, we speculate that the influence of *F. nucleatum* on the polarization of macrophages depends on the microenvironment, where the macrophages are located. Our hypothesis is that the infection with *F. nucleatum* provides an exacerbated inflammatory response via the NLRP3 inflammasome or excessive autophagy that influences the microbiota environment and favors CRC initiation and progression. In terms of disease control strategies, we aim to use newly synthesized NLRP3 inhibitors or autophagy blockers to reverse the harmful effects of *F. nucleatum* infection on CRC. The specific aims of the project are listed in Table 1 together with the related research units.

## 4. Methods and Analysis

### 4.1. Experimental Design (ED) for Aim 1

To address our hypothesis and dissect the molecular mechanisms underlying the inflammatory and pro-tumorigenic actions of *F. nucleatum*, we have designed a series of in vitro experiments. These studies will explore how different *F. nucleatum* strains affect epithelial and immune cells, with particular focus on NLRP3 activation and downstream inflammatory responses. We will infect macrophages or epithelial cells and CRC cells with *F. nucleatum* and analyze the NLRP3 activation by ELISA and immunoblot assays [25]. Specifically, in line with the literature [26], we will analyze three bacterial strains of *F. nucleatum*, derived from three different anatomical isolations: EAVG_005 (13_3C) from the ascending colon, EAVG_023 (3_2_44B) derived from the descending colon, and EAVG_027 (2_1_50A) derived from the more distal colon/rectum. Additionally, the human leukemic cell line THP-1 will be obtained from ATCC. For cell proliferation, the cells will grow in T-25 flasks with an initial cell count of 2 million cells in a volume of 5 mL. To differentiate THP-1 into THP-1 macrophages (Thp-1m) for our experiment, we will seed the cells at densities of 2 × 10^5^ and 1 × 10^6^ cells/well in six-well plates. PMA will be evaluated at doses of 50, 100, and 200 ng/mL. After 48 h of PMA stimulation, THP-1m will be obtained. After differentiation, all THP-1m cells will be washed out of the PMA-containing medium and cultured in fresh CGM without PMA for 21 days. Two CGM recipes for long-term culture will be used, namely, RPMI-1640 and DMEM (HyClone, Cytiva, Marlborough, MA, USA) containing the same supplements of 10% heat-inactivated FBS, 100 units/mL penicillin–streptomycin, and 2 mM L-glutamine. The human intestinal epithelial cell line, HIEC-6 (ATCC), will be grown in 50% DMEM and 50% Ham’s F12 Nutrient Mixture (Merck, Darmstadt, Germany). Relatively to CRC cells, the human epithelial colon cancer cell lines HT-29 and HCT116 will be purchased from ATCC. To initiate co-cultures of macrophages or epithelial cells and CRC cells with *F. nucleatum* strains, liquid cultures of *F. nucleatum* will be pelleted down, the supernatant will be discarded, pellets will be resuspended in the same volume of Brain Heart Infusion (BHI) broth, and 4 μL of suspension will transferred to each well of the microplate (Multiplicity Of Infections (MOIs) of approximately 1.8:1–0.4:1, depending on the literature). This MOI is based on previously published in vitro studies involving *F. nucleatum* co-culture with human epithelial and immune cells [25,26]. This MOI range has been shown to activate inflammatory pathways, including NLRP3 inflammasome signaling, without causing excessive cell death that could compromise downstream mechanistic analyses. To ensure the reproducibility and validity of our co-culture conditions, bacterial viability will be routinely assessed through colony-forming unit (CFU) counts. Prior to co-culture, *F. nucleatum* cultures will be grown anaerobically, and bacterial suspensions will be adjusted to the appropriate optical density (OD600). Serial dilutions will then be plated on selective media to confirm CFU/mL and calibrate the inoculum. This approach allows us to standardize bacterial input across experiments and confirm viability prior to infection. Co-cultures will then be incubated at 37  °C, 5% CO_2_ (standard humidified incubator). After exposure of human cells with *F. nucleatum*, NLRP3, IL-1, IL-6, IL-8, and CXCL-12 will be quantified in human cell cytoplasm through selective ELISA methods.

To assess a possible correlation between an inflammatory status induced by *F. nucleatum* and a pre-cancerous lesion into the intestinal epithelium, we will establish a co-culture model. Macrophages, as the primary releasing cells, will be seeded into a removable insert and activated with different amounts or species of *F. nucleatum*. On the bottom of the plate, we will place epithelial cells: possible target cells whose function could be altered by an inflammatory response. Analysis of cell proliferation, autophagy induction, and metabolism of epithelial cells will be performed, as well as foci forming assays. To ascertain whether a sustained inflammatory response mediated by altered microbial colonization may favor tumor progression, we will evaluate the direct effect of inflammatory cytokines released by macrophages on cancer cells. The proliferation, migration, and invasion of different types of CRC cells cultured alone in the presence of *F. nucleatum* or in co-culture with macrophages or other cells that trigger NLRP3 activation following infection with *F. nucleatum* will be evaluated. To explore the role of *F. nucleatum* in mediating chemoresistance, possible through the induction of an inflammatory response and autophagy modulation, we will treat primary or metastatic colon cancer cells with different anti-cancer drugs. CRC cell lines, as well as primary CRC cells isolated from patients’ tumor tissues, will be used as models. We will verify the cytotoxic effects of a panel of anti-cancer drugs (including 5-FU, platinum-based compounds, irinotecan, anti-VEGFR agents, and anti-EGFR therapies) on those cells. Cell viability, cell death, and metabolism of the cell models will be evaluated during different concentrations of drug exposure in the presence or absence of the bacterium *F. nucleatum*. Subsequently, we will co-culture macrophages on the top and CRC cells on the bottom and analyze the effect of anti-cancer drugs in combination with *F. nucleatum*, to discriminate the effect mediated by inflammation. Mechanisms of chemo- and immune resistance will be analyzed in cytoplasm extracts of colon cancer cells at the end of treatments through quantification of Myddosome (Myd-88), NLRP3 inflammasome, and several other cytokines/growth factors (IL-1a, IL-1ß, IL-2, IL-4, IL-6, IL-10, IL-12, IL17-a, IFN-a, TNF-a, G-CSF, and GM-CSF). Finally, to investigate the molecular mechanisms involving *F. nucleatum* in the development of NLRP3-mediated and autophagy-dependent chronic inflammatory environments, we will use modulators specifically targeting these pathways. The effects of newly synthesized NLRP3 inhibitors [27] and autophagy inhibitors such as chloroquine (CQ), at different concentrations and time points, will be evaluated in counteracting *F. nucleatum* consequences in in vitro models of transformation, CRC progression, and chemoresistance.

### 4.2. Experimental Design for Aim 2

To validate and extend our in vitro findings in a clinically relevant context, we will conduct a translational study involving patients affected by IBD and CRC. This will allow us to correlate microbial signatures and inflammatory profiles with disease status and clinical features. Patients of any ethnicity, who are at least 18 years old with a diagnosis of IBD and CRC not previously treated, are eligible to participate. The project will enroll patients in two different arms: healthy subjects will represent the control group, whereas subjects affected by IBD or CRC will represent the study group. The inclusion and exclusion criteria are listed in Table 2.

Subjects undergoing a pan-colonoscopy at the Endoscopy Digestive Unit of the University Hospital “G. Martino” of Messina for screening with or without a positive fecal occult blood test or suspicion of IBD or CRC will be evaluated for the inclusion. Normal tissues and tumor tissues, where present, and biological fluids will be collected from all patients. Recruiting physicians will determine whether a subject is eligible for participation and note all subjects, including their age and confirmed diagnosis (IBD or CRC). A total of 150 subjects will be enrolled from a population (target population) with a diagnosis of IBD and CRC and healthy subjects.

One week before the colonoscopy, fecal samples will be collected from all patients and stored at +4 °C. For each patient, a colon biopsy will be performed through a colonoscopy. In collaboration with the Pathological Anatomy Unit of Messina University, tissues fixed in 4% paraformaldehyde will be embedded in paraffin, sectioned, and then stained with Hematoxylin and Eosin (H&E). The morphological changes will be examined under a microscope equipped with a digital camera. Immunohistochemistry (IHC) will be applied to determine the expressions of NLRP3, IL1, IL-8, CXCL-12, and IL-6 in tissue samples gained from patients. Then, the sections will be blocked by 3% hydrogen peroxide, followed by incubation at 4 °C overnight with rabbit anti-NLRP3 or anti-IL1, -IL-8, -CXCL-12, or anti-IL-6. Colon and fecal samples will be sent to the Division of Microbiology of the University of Messina for the research of *F. nucleatum* and the complete characterization of the intestinal microbiota. IHC will be used to localize and quantify the expression of key inflammatory and tumor-related markers directly within colonic tissue sections from patients. This technique allows us to assess not only the presence but also the spatial distribution of these proteins in different tissue contexts (healthy, IBD, and CRC). IHC provides critical information on the activation state of inflammatory pathways and their association with epithelial transformation, which is essential for correlating local immune responses with the presence of *F. nucleatum* and disease progression. These data will help to define a tissue-specific inflammatory signature and identify potential biomarkers of prognosis or therapeutic resistance, which will then be correlated with microbiome profiling, cytokine analyses, and radiomic features. Venous blood samples will be collected from patients to obtain plasma and serum. All samples will be sent to the University of Ferrara to analyze cytokines and systemic growth factors through the ELLA platform.

MDA and 4-HNE levels will be measured in stool supernatants (as surrogate indicators of luminal oxidative stress). MDA will be assessed by a colorimetric thiobarbituric acid-reactive substances (TBARSs) assay, following lipid extraction and protein normalization. This method is sensitive and routinely applied in clinical and translational oxidative stress studies. 4-HNE will be measured by ELISA using commercially available competitive immunoassay kits (e.g. Abcam plc, Cambridge, UK or Elabscience Biotechnology Inc., Houston, TX, USA.

Patients with a confirmed diagnosis of CRC will undergo computed tomography (CT) staging. CT images will be performed at time 0, and then every 3 to 6 months depending on the stage of the disease. The acquisition of CT images will be performed with a standardized protocol. The CT images will be shared with the UO2 AULSS 9 Scaligera for the radiomic analysis (see aim 3). The colon sample tissue of the subjects will be analyzed for the following information: (a) confirmation of diagnosis using healthy tissue versus IBD versus CRC; (b) the analysis of the microbiome profile; (c) prognostic factors in subjects with IBD and CRC and according to the microbiome profile; (d) role of *F. nucleatum* as a prognostic factor in IBD and CRC; and (e) *F. nucleatum* as a key role in the pharmacoresistant process in the treatment of CRC. IHC analysis from paraffin-embedded sections against pro-inflammatory cytokines and novel biomarkers of cancer risk and prognosis will be performed. These data will be matched with the data obtained from the biological analyses performed by the aim 1 component to define an inflammatory profile for each patient. Moreover, correlative analyses will be performed to relate the bacterial amount and species to (i) the clinical characteristics of enrolled patients, (ii) the patient inflammatory status, and (iii) the possible chemoresistance developed by some of them.

### 4.3. Experimental Design for Aim 3

Finally, to explore the potential of imaging biomarkers as non-invasive indicators of inflammation and *F. nucleatum*-related changes, we will integrate a radiomic approach. By linking imaging features with microbial and molecular data, we aim to develop a predictive model for patient stratification and clinical outcomes. In this study, we will use a virtual biopsy technique. Firstly, we will extrapolate a predictive model for the presence or absence of chronic inflammation using retrospective series and clinical outcomes. Patients will be stratified into different risk groups based on TNM staging and histology. To identify patients at different risks despite having the same histology and clinical features, a time-to-event analysis will be performed. Overall survival (OS) and disease-free survival (DFS) will be analyzed using a time-to-event analysis approach. CT abdomen images will be manually exported and stored in a dedicated Picture Archiving and Communication System (PACS). To ensure consistency in sampled volume for radiomic feature extraction, we will employ a “virtual biopsy” technique by drawing sampling spheres with a fixed diameter of 0.5 cm. Each cancer volume will have one sphere centered on the core of the lesion, and two additional spheres will be placed at the tumor perimeter using different 3D contouring approaches to analyze the tumor burden and peritumoral region. Spheres will be drawn on the axial and coronal planes of the slice showing the largest lesional diameter, respectively [28]. Spheres of the same diameter will also be placed in three regions of the colon to explore the inflammation status of the colon. From each delineated volume of interest (VOI), a wide range of radiomic features will be extracted, including morphological features, intensity-based statistical features, intensity histogram features, texture matrix-based features with IBSI-consistent implementation, different feature aggregation methods, and high-order statistical features. The Medical Image Merge (MIM) (MIM Software (version 7.1.6, MIM Software Inc., Cleveland, OH, USA) and Pinnacle software (Pinnacle^3^,version 16.2, Philips Radiation Oncology Systems, Fitchburg, WI, USA) will be used for the segmentation of CRC lesions (virtual biopsy) and the colon at three points: near the ileocecal valve, the transverse colon (middle point), and the descending colon before the sigmoid. Radiomic features will be extracted using Life X 7.14 software. All patient CT images (staging, abdominal CT, and follow-up CT) will be transferred from Messina, stored in a dedicated PACS, and thus analyzed following the methods described. If a relationship between the amount of *F. nucleatum*, NLRP3-dependent inflammation, and the clinical characteristics of the patients is demonstrated, the same relationship will be evaluated using radiomics.

### 4.4. Methodologies and Statistical Analyses

#### 4.4.1. Materials and Methods of Data Collection

For the purpose of the project, several cell lines will be used: Human Colon Epithelial Cells (HCnEpC), THP-1, and colorectal cell lines derived from normal colon mucosa (Human epithelial colon NCM460 cells) or from tumors at various levels of differentiation and stages of development (CACO-2 moderately well-differentiated adenocarcinomas consistent with colonic primary grade II, Human adenocarcinoma cell lines, HT-29, HCT-116, and SW480, and the corresponding lymph node metastatic SW620 cell line). Moreover, three *F. nucleatum* subspecies will be used, including FNP (ATCC 10953), FNN (ATCC 25586), and FNV (ATCC 49256), and will grow on a BHI agar plate and Tryptic Soy Broth (TSB) (1.55% BHI; 1.48% TSB; 1.7% agar; 0.5% yeast extract; 1% menadione solution (0.5 mg/mL); 1% hemin solution (1 mg/mL); and 5% defibrinated sheep blood) in an anaerobic jar system with Anaerocult A anaerobic generators for 7 days at 37 °C. Bacterial quantification was performed using spectrophotometry at 550 nm. Cells will be treated with different concentrations of oxaliplatin (1–100 μM) and 5-FU (1–100 μM) irinotecan, anti-VEGF monoclonal antibody, and anti-EGFR monoclonal antibodies for 24, 48, and 72 h. Pre-tests will involve treating cells with increasing concentrations of each drug, followed by assessment of cell viability using standard assays (such as MTT or CellTiter-Glo). Sub-lethal doses (typically ≤ IC30) will then be selected for downstream experiments, in order to minimize unspecific cytotoxic effects and more accurately model clinically relevant drug exposures. Concerning NLRP3 inhibitors, compounds 6c, 7n, and 10, with high potency and selectivity in inhibiting NLRP3 activation in vitro, will be used at a concentration of 1 μM [27]. Related to autophagy inhibitors, CQ and Bafilomycin A1 will be used at 0.1, 1, 10, 100, or 1000 μM CQ for 12 and 24 h, to determine the dose of CQ that effectively inhibits autophagy without affecting the proliferative activity of colon cells. In the analysis of NLRP3 activation, IL-1ß and IL-18 secretion will be quantified by ELLA (Simple Plex Explorer software version 4.1.0.22, R&D Systems, Bio-Techne, Minneapolis, MN, USA) according to the manufacturer’s instructions, and by Western blotting. Cell migration will be evaluated using Culture-Insert 2 Well in µ-Dish 35 mm (Ibidi) in the presence or absence of vehicle or chemotherapics in combination with NLRP3 inhibitors or autophagy blockers. Acquisitions will be performed at 0, 24, 48, and 72 h. Apoptosis will be determined by three different methods: (i) by blotting for different cell death markers, such as cleaved PARP and cleaved CASPASE-3, NF-kB, and the downstream target gene products Bcl-2, Bcl-XL (BCL2L1), and XIAP15; (ii) by RealTime-Glo Annexin V Apoptosis and Necrosis Assay (Promega); and (iii) by automated nuclei count analysis, as described [29]. Cells will be seeded in XF96 Cell Culture Microplates. The oxygen consumption rate (OCR) and extracellular acidification rate (ECAR) will be measured simultaneously using the XF96 Analyzer following the manufacturer’s instructions (Seahorse Biosciences). For the chemosensitivity analysis, cancer cells will be plated in multi-well plates and exposed to different concentrations of the selected drugs. Cell viability will be assessed using various methods [30]. A database will be created to organize the data collected from the patients, assigning a sequential ID to each patient. The patient samples will be categorized into three groups: healthy, IBD, and CRC. As for the ID format, a combination of letters and numbers to represent each category will be used. For example: healthy patients (H1, H2, and H3), IBD patients (B1, B2, and B3), and CRC patients (C1, C2, and C3). To further differentiate the sample types, each collected sample will be labeled accordingly. For example, H1f will represent a fecal sample from healthy patient 1, while H1b will represent a blood sample from the same patient. The database will then be shared with all four research units involved in the study, allowing each unit to contribute and analyze their respective data within the shared framework. Additionally, a second database will be created for the in vitro data. Data segregation into distinct databases will facilitate organization and analysis, ensuring efficient management of both clinical and in vitro data within the study and the research units.

#### 4.4.2. Statistic Plan

For the in vitro studies, a minimum of three replicates for each experiment will be performed to ensure reliable and statistically significant results. The serum samples obtained from the patients will be collected and then analyzed for cytokine release by ELISA. During the analysis, each sample will be read in triplicate. Subjects undergoing full colonoscopy at the Endoscopy Unit of the University Hospital “G. Martino” in Messina, either for routine CRC screening (with or without positive fecal occult blood test) or for clinical suspicion of IBD or CRC, will be screened for study eligibility. For each participant, matched healthy mucosa, tumor tissue (where applicable), and biological fluids (e.g., blood, saliva, and stool) will be collected. Clinical data including age and confirmed diagnosis (healthy, IBD, or CRC) will be recorded. A total of 150 subjects will be enrolled, equally distributed across three groups: healthy controls (*n* = 50); IBD patients (*n* = 50); and CRC patients (*n* = 50).

The sample size for this study was established based on a set of statistical assumptions and methodological considerations aimed at ensuring sufficient power to detect biologically meaningful differences among groups. The primary objective is to demonstrate a statistically significant difference in the mean concentration of pro-inflammatory cytokines across the three clinical cohorts under investigation: healthy individuals, patients with IBD, and those with CRC. To this end, the analysis is designed to detect a minimum intergroup difference equivalent to 0.5 standard deviations in cytokine levels, which corresponds to a moderate effect size (Cohen’s d = 0.5). Comparisons among the three groups will be performed using one-way ANOVA, followed by pairwise post hoc tests adjusted for multiple comparisons (e.g., Bonferroni correction), where appropriate. The statistical model assumes that cytokine concentrations are approximately normally distributed and that the variance is homogeneous across groups, although these assumptions will be empirically assessed and, if necessary, addressed using data transformations or nonparametric methods. The significance threshold for hypothesis testing has been set at α = 0.05 (two-tailed), with a corresponding Type II error probability of β = 0.10, resulting in a statistical power of 90%. Under these assumptions, a total sample size of 150 participants—distributed equally across the three groups (*n* = 50 per group)—is deemed sufficient to detect the expected differences with the specified level of confidence. This estimation was derived from standard power calculations for ANOVA-based comparisons and ensures that the study is appropriately powered to detect moderate biological effects in cytokine expression.

#### 4.4.3. Statistical Analysis

The statistical analysis for the specific aims of the project will be as follows.

Aim 1: For the in vitro studies, all data will be analyzed by Prism 6 (GraphPad, San Diego, CA, USA). Two-group datasets will be analyzed by Student’s unpaired *t*-test. For three or more group analyses, one-way ANOVA Tukey’s multiple comparison tests will be used. Linear regression analysis will be performed with the GraphPad Prism 7.02 software x2 test.

Aim 2: The data will be analyzed by Prism 6 (GraphPad). Normal distributions will be assessed using the Shapiro–Wilk test. Student’s t-test for unpaired data with Welch’s correction will be used to compare two datasets with normal distributions, while the Mann–Whitney test will be used for non-normal datasets. Descriptive analysis of demographic, clinical, and pathological data will be performed assessing the mean (standard deviation, SD) values. Categorical variables will be described as counts and percentages. *p*< 0.05 will indicate statistical significance.

Aim 3: All radiomics quantitative parameters will be reported as a median value and interquartile range (IQR). Data from both patient groups will be analyzed using the statistical R software (version 4.3.1, R Foundation for Statistical Computing, Vienna, Austria) and the package RadAR (version 1.1.0, Radiomics Analysis With R). To perform feature selection, the Minimum redundancy–maximum relevance (mRMR) method will be adopted. We would like to define differential radiomics with the goal of identifying statistically different radiomic features between groups of patients. These groups will be defined based on the results of aim 2. The Mann–Whitney U test and the area under the curve (AUC) will be used for two-group comparisons, and the Kruskal–Wallis test will be used for comparisons between three or more groups.

## 5. Discussion

Growing evidence suggests a significant connection between chronic inflammation, specific bacterial infections, and an increased risk of developing cancer [5]. The human microbiota primarily provides numerous benefits to the host [2]; however, dysbiosis, characterized by changes in bacterial populations or their metabolic products, has been increasingly implicated in the pathogenesis of CRC. Among the most studied microorganisms, *F. nucleatum* has emerged as a key gut environmental factor that can modulate both CRC development and recurrence through drug resistance mechanisms [14,15].

The intestinal microbiota dysbiosis mediated by *F. nucleatum* influences CRC risk through the activation of chronic inflammation pathways. Inflammation is a natural immune response to tissue damage or infection; however, prolonged inflammation can promote carcinogenesis. Moreover, *F. nucleatum* has been linked to the activation of autophagy and inflammasome pathways, both of which are associated with chemoresistance in CRC cells [5,9,10]. This dual role, either supporting or suppressing tumorigenesis depending on context, makes microbial manipulation a potentially useful strategy for cancer prevention and treatment, though further mechanistic insight is still needed [16,17,18].

Recent studies have revealed that *F. nucleatum* can activate the NLRP3 inflammasome via its virulence factor FadA, leading to IL-1β secretion and chronic intestinal inflammation. This inflammatory cascade is tightly interconnected with the overproduction of ROS, creating a redox–inflammatory loop that drives epithelial damage and genomic instability [31]. ROS not only contribute to inflammation but also initiate lipid peroxidation, leading to the formation of bioactive aldehydes such as MDA and 4-HNA. These compounds form adducts with proteins, lipids, and DNA, disrupting cell function and promoting carcinogenesis. Elevated tissue levels of MDA and 4-HNA are associated with disease severity in both IBD and CRC, suggesting their potential as biomarkers of oxidative stress and disease progression [32]. This redox-driven axis, linking microbial dysbiosis, inflammation, and oxidative damage, contributes to neoplastic transformation and could serve as a target for therapeutic intervention.

The mechanistic pathway linking *F. nucleatum* to cancer involves adhesion to epithelial cells, followed by internalization and activation of TLR4/MYD88 signaling [33]. This stimulates NF-κB-mediated transcription of pro-inflammatory cytokines and contributes to mitochondrial ROS production, further amplifying NLRP3 inflammasome activation and reinforcing the inflammatory state of the tumor microenvironment.

Importantly, ROS can act as secondary messengers to further upregulate NLRP3 expression, perpetuating a self-sustaining cycle of oxidative stress and inflammation. This complex interaction positions *F. nucleatum* as a central modulator of both immune and redox responses in CRC pathogenesis.

According to the importance of bacterial metabolites in gut homeostasis and carcinogenesis, modulating the microbiota composition or function, either by direct antimicrobial strategies or microbiome-focused therapies, represents a promising adjunct in cancer management. The connection between inflammation, microbial dysbiosis, and cancer progression has significant implications for clinical practice, especially in the context of personalized prevention and early detection.

From a translational perspective, several applications could be envisioned:

- Targeted screening for *F. nucleatum* in fecal samples or biopsy tissue could help identify individuals at elevated risk of CRC.
- Monitoring ROS-related lipid peroxidation markers (e.g., MDA and 4-HNA) in stool, plasma, or tissue may offer a non-invasive means to assess oxidative stress and disease progression.
- Patients with IBD or recurrent CRC might particularly benefit from microbiota profiling and redox biomarker assessment, guiding tailored prevention and surveillance strategies.

Furthermore, radiomics-based imaging may enhance risk stratification by correlating tissue inflammation, autophagy activation, and microbial signatures, potentially serving as a predictive tool for CRC onset and progression.

The study also opens new avenues for investigating mechanisms of chemotherapy and immunotherapy resistance linked to microbial modulation. By integrating molecular and clinical data, this research may help guide more effective drug selection, particularly in patients harboring a pro-inflammatory microbiome profile.

Finally, the identification of dysregulated pathways such as NLRP3 activation and autophagy in pre-cancerous and cancerous conditions may inform the development of novel therapeutic strategies, including NLRP3 inhibitors and autophagy modulators [27]. A comprehensive understanding of the interplay between the microbiome and host immune responses holds great potential for advancing CRC prevention, diagnosis, and treatment.

However, despite the promising potential of bacterial biomarkers such as *F. nucleatum* in CRC and IBD prognostics and diagnostics, several challenges must be considered. First, the complexity and variability of the gut microbiome across individuals, influenced by diet, geography, genetics, and medication, complicate the establishment of universal microbial signatures. Second, bacterial detection methods vary in sensitivity and specificity, and sample heterogeneity (e.g., fecal vs. tissue biopsy) may affect reproducibility and clinical interpretation. Third, the dynamic nature of the microbiome requires longitudinal monitoring to accurately capture disease-related changes rather than transient fluctuations. Finally, microbial markers must be integrated with host genetic, epigenetic, and environmental factors to improve predictive accuracy.

## 6. Conclusions

The research highlights the profound associations between chronic inflammation, lipid peroxidation, microbial dysbiosis, and cancer, particularly CRC. Identifying changes in the gut microbiota may contribute to the development and implementation of precise diagnostic tools, biomarkers, and screening strategies to detect cancer in its early stages, when treatment outcomes are generally more effective. The study underscores the importance of early detection and treatment of bacterial infections and inflammation to reduce the risk of developing CRC and paves the way for innovative approaches to target *F. nucleatum* infections. Early diagnosis and intervention not only improve patient outcomes but also increase cost-effectiveness by reducing the economic burden associated with advanced-stage cancers. This research highlights the importance of considering the relationship between the microbiome and cancer in public health initiatives and clinical practice to optimize patient care and resource allocation.

## Figures and Tables

**Table 1:** Specific aims and research units of the study.

	Specific Aim	Research Unit
Aim 1	To characterize the in vitro relevance of *F. nucleatum* infection in NLRP3 inflammasome activation and associated cytokines, autophagy induction, CRC development and progression, and cancer cell resistance to chemotherapy	Division of Cardiology, Istituto Nazionale Tumori- IRCCS Fondazione Pascale Naples Department of Medical Sciences, University of Ferrara
Aim 2	To define the association of *F. nucleatum* with chronic IBD and CRCs to reveal possible novel *F. nucleatum*-related biomarkers for early screening purposes	Endoscopy Digestive Unit, Department of Clinical and Experimental Medicine, Policlinico “G. Martino”, University of Messina
Aim 3	To demonstrate the potential of radiomics for the identification of specific prognostic imaging phenotypes in IBD and CRC patients to uncover the relationship between quantitative image features and physio-pathological data, specifically focusing on radiological biomarkers related to tumor/host interaction and the tumor microenvironment (TME)	Department of Clinical Oncology, AULSS 9 Scaligera, Verona

**Table 2:** Inclusion and exclusion criteria for specific aim 2.

Inclusion Criteria	Exclusion Criteria
Subject must be over 18 years old	Subjects previously treated for IBD and CRC
Healthy subject (50 pts)	Previous exposure to antibiotic therapy and/or corticosteroid treatments (4 months before the enrollment)
Subject affected by IBD (50 pts)	Life expectancy is estimated to be less than three months for CRC subjects
Subject affected by CRC (50 pts)	Subjects with a previous diagnosis of cancer
Subject has signed informed consent	Subjects unwilling to take part
	Subject is, in the opinion of the Investigator, not suitable to participate in the study

## Data Availability

No new data were created or analyzed in this study. Data sharing is not applicable to this article.
